# Unveiling Dietary Complexity: A Scoping Review and Reporting Guidance for Network Analysis in Dietary Pattern Research

**DOI:** 10.3390/nu17203261

**Published:** 2025-10-17

**Authors:** Rebecca M. J. Taylor, Jack A. Moore, Amy R. Griffiths, Alecia L. Cousins, Hayley A. Young

**Affiliations:** School of Psychology, Faculty of Medicine, Health, and Life Science, Swansea University, Wales SA2 8PP, UK; 1905477@swansea.ac.uk (R.M.J.T.); 1911306@swansea.ac.uk (J.A.M.); a.r.griffiths@swansea.ac.uk (A.R.G.); a.l.cousins@swansea.ac.uk (A.L.C.)

**Keywords:** dietary patterns, models, statistical

## Abstract

**Background/Objectives**: Dietary patterns play a crucial role in health, yet most research examines foods individually, overlooking how they interact. This approach provides an incomplete picture of how diet influences health outcomes. Network analysis (e.g., Gaussian graphical models, mutual information networks, mixed graphical models) offers a more comprehensive way to study food co-consumption by capturing complex relationships between dietary components. However, while researchers have applied various network algorithms to explore food co-consumption, inconsistencies in methodology, incorrect application of algorithms, and varying results have made interpretation challenging. The objectives of this scoping review were to systematically map and synthesise studies that have applied network analysis to dietary data, and to establish guiding principles for future research in this area. **Methods**: Using PRISMA-ScR criteria, our scoping review identified 171 articles published from inception up to 7 March 2025, of which 18 studies met the inclusion criteria. **Results**: Gaussian graphical models were the most frequent approach, used in 61% of studies, and were often paired with regularisation techniques (e.g., graphical LASSO) to improve clarity (93%). The analysis revealed significant methodological challenges across the literature: 72% of studies employed centrality metrics without acknowledging their limitations, there was an overreliance on cross-sectional data limiting the ability to determine cause and effect, and difficulties in handling non-normal data. While most studies using GGM addressed the issue of non-normal data, either by using the nonparametric extension, Semiparametric Gaussian copula graphical model (SGCGM), or log-transforming the data, 36% did nothing to manage their non-normal data. **Conclusions**: To improve the reliability of network analysis in dietary research, this review proposes five guiding principles: model justification, design–question alignment, transparent estimation, cautious metric interpretation, and robust handling of non-normal data. To facilitate their adoption, a CONSORT-style checklist is introduced—the Minimal Reporting Standard for Dietary Networks (MRS-DN)—to help guide future studies. This review was preregistered on Open Science Framework.

## 1. Introduction

Dietary patterns have been associated with a variety of health outcomes. For example, the Mediterranean diet, characterised by a high consumption of fruits, vegetables, whole grains, and healthy fats, has been linked to the prevention of cardiovascular disease [1,2], better cognitive performance [3], and a longer life expectancy [4]. In comparison, the Western diet, consisting of a high intake of red and processed meat, refined grains, sugars, fats, and fast food with a low intake of fruit and vegetables, has been associated with higher rates of obesity [5,6] and an increase in cancer risk [7].

Currently, most of the research looking into nutrition and its effect on health has focused on analysing foods and nutrients separately from each other [8,9] or has produced “a priori” diet quality scores or “data-driven” composite scores [10]. These traditional dietary assessment methods do not expose food synergies, which may lead to an incomplete understanding of dietary patterns and their health implications [11,12]. Therefore, it is crucial to research not only what foods are consumed but also how foods are consumed in combination. For example, a recent study found that garlic may counteract some of the detrimental effects associated with red meat consumption, including a reduced risk of cardiovascular disease from a high intake of red meat [13]. This finding emphasises the need to examine the interactions between different foods to fully understand their health impacts.

The network approach offers a promising and more holistic way to analyse the co-consumption of foods. It enables the exploration of dietary patterns by using advanced statistical techniques to map and analyse the connections between various dietary components [12]. By capturing the co-consumption patterns and their associated outcomes, the network approach can reveal insights into the relationship between nutrition and health that traditional methods may have previously overlooked.

### 1.1. Dietary Patterns and Health

Using dietary analysis in nutritional research is essential for the development of dietary interventions aimed at improving both physical and psychological wellbeing. Accurate dietary analysis allows researchers to identify dietary patterns and nutrients that influence health outcomes, enabling the formulation of targeted interventions. For instance, the Dietary Approach to Stop Hypertension (DASH) diet was developed after observational research found that a carbohydrate rich diet with fruits, vegetables, and low-fat dairy products was associated with lowered blood pressure [14,15]. Notably, randomised controlled trials of individual nutrients such as magnesium, potassium, calcium, and fibre had produced inconsistent results [14]. One explanation was that nutrients from dietary supplements may not benefit health as effectively as those obtained from whole foods due to the synergistic interactions between nutrients and other components present in the diet. Unfortunately, due to the limitations of conventional dietary pattern analysis, most nutritional interactions remain undiscovered. However, recent computational advances may help unveil nonadditive and nonlinear interactions [16], thereby improving dietary recommendations and the development of multicomponent functional foods and supplements that benefit health [8].

### 1.2. Traditional Dietary Pattern Analysis

The traditional methods used for dietary pattern analysis include principal component analysis (PCA), cluster analysis, and a priori composite scores. As detailed in Table 1, these techniques are used to summarise complex dietary data into more easily interpretable patterns/groups. For instance, PCA might identify a “healthy eating” component by grouping correlated foods like fruits and vegetables [17,18], while cluster analysis may group individuals with similar overall diets [19]. These methods have been instrumental in linking broad dietary patterns, such as the Western diet, to adverse health outcomes, such as obesity [5,20] and cancer [21].

Despite their utility and valuable insights, these traditional methods share a significant limitation—they are often unable to fully capture the complex interactions and synergies between different dietary components [22]. By reducing dietary intake to composite scores or broad patterns, the multidimensional nature of diet is often disregarded, and crucial food synergies may be hidden [23]. While these patterns may capture some synergies, this is only possible when interactions are explicitly recognised and incorporated during score development, which is rare [24]. Moreover, in research focusing on individual nutrients or foods, interactions are often implicitly assumed to be nonexistent in the model design [24]. Finally, these methods often assume that dietary patterns are relatively static, ignoring potential changes in diet over time due to ageing, economic changes, or health conditions [25]. These incorrect assumptions about interactions, or assumptions of staticity in model design, can result in obscured or false associations and biased effect estimates.

In contrast, for the methods usually used to quantify dietary patterns, more prescriptive approaches such as linear optimisation have been instrumental in designing diets that meet specific targets for health, sustainability, or cultural appropriateness. Whilst beyond the scope of this review, these methods are fundamentally “knowledge-based”, meaning they can only optimise for the variables they already know about—the handful of macro- and micronutrients that have been well characterised. This represents the “known knowns” of nutrition, which constitute less than 1% of the thousands of distinct bioactive compounds and phytochemicals present in our food chain. This inherent limitation means that such models are blind to the vast “nutritional dark matter” and the complex food synergies that are crucial for health.

### 1.3. Network Approaches

To overcome the limitations of traditional dietary analysis, network approaches have emerged as a promising alternative, representing a superior, bottom-up alternative to knowledge-based prescriptive models like linear optimisation. Unlike the traditional methods which focus on individual nutrients and patterns in isolation [26], network analysis does not require comprehensive prior knowledge of every bioactive compound. Instead, it is a data-driven approach that learns directly from real-world eating behaviours. While also data-driven, this provides a key advantage over methods like PCA or cluster analysis; instead of reducing diet to composite scores or groups, network analysis explicitly maps the web of interactions and conditional dependencies between individual foods [12].

Methods such as Gaussian graphical models (GGMs) and mutual information (MI) networks enable researchers to visualise and analyse these intricate relationships within a diet [27]. By mapping the connections between foods and nutrients, these methods reveal how they collectively influence health outcomes and allow for the discovery of beneficial food combinations and protective synergies that emerge rather than a pre-defined biochemical model. Furthermore, dynamic or time-varying networks can model how dietary patterns change over time within individuals or populations, turning the complexity of our diet from a limitation into a source of discovery [28].

A variety of network algorithms have been developed, although not all have hitherto been applied to diet (Table 2). GGMs are probabilistic models that use partial correlations to identify conditional independence between variables. These models are particularly useful for exploring linear relationships in dietary data, offering insights into how one nutrient interacts with others while accounting for the broader dietary context. For example, GGMs can reveal whether the intake of saturated fats and sodium is conditionally independent given calorie consumption. This could help identify whether their relationship is direct or merely a byproduct of consuming high-calorie foods. This makes them valuable for understanding direct and indirect nutrient associations within diets. A limitation is that GGMs assume linear relationships, making them unsuitable for capturing the nonlinear interactions that are often present in dietary data. For example, the effect of salt on hypertension may be moderated by the potassium and sugar content of the diet [29]. Additionally, GGMs are sensitive to non-normal distributions, which can distort the results in datasets with significant deviation [30].

Related to GGMs, mixed graphical models (MGMs) accommodate datasets containing both continuous variables (e.g., nutrient intake) and categorical variables (e.g., demographic characteristics) [31]. This versatility is particularly useful for dietary studies that integrate diverse types of information. For example, MGMs can explore how continuous measures of dietary intake correlate with categorical socioeconomic factors such as education or income. By modelling these mixed data types jointly, MGMs expand the applicability of graphical models to more complex nutritional datasets, potentially yielding deeper insights into diet-health relationships. However, MGMs share several limitations with GGMs, including sensitivity to non-normal distributions for continuous variables [30].

MI networks measure the amount of information shared between pairs of dietary components, capturing both linear and nonlinear associations [32]. This can uncover hidden patterns and relationships that may not have been found using traditional correlation-based methods [33]. For instance, by modelling nonlinear patterns, MI networks can explore how sugar and fat intake interact to disproportionally influence obesity or cardiovascular risk, identifying subtle dependencies such as threshold effects that might be missed by simpler models. However, a significant limitation is that MI algorithms usually give rise to denser networks reducing interpretability and the ability to tease apart direct and indirect dependencies.

Bayesian networks (BNs) are probabilistic graphical models that represent the relationships between variables through directed acyclic graphs, enabling the identification of potential causal pathways [34]. BNs have not yet been applied to dietary pattern analysis; however, unlike other traditional correlation-based methods, BNs provide insights into causality [34] which may make them a powerful tool to explore how changes in dietary components may influence one another. One possible application of BNs is to model the fat–sugar seesaw phenomenon, where reducing fat tends to lead to an increase in sugar in the diet [35]. One advantage of BNs is their ability to incorporate prior knowledge into the model structure [36], potentially enhancing the interpretability and plausibility of the derived dietary network. There are limitations to BNs, in particular the computational difficulty of exploring a previously unknown network [37].

Dynamic networks incorporate time-varying dependencies [38], enabling researchers to observe how dietary patterns and meal compositions evolve over time. This approach allows the study of how diets respond to external factors, such as seasonal changes or price increases, and how interventions might alter established dietary habits [39]. One possible application of dynamic networks is to understand how patterns of co-consumption may change following dietary interventions. For example, if a person tends to consume meat with vegetables and an intervention reduces meat consumption, dynamic networks could reveal whether this intervention also leads to an unintended reduction in vegetable intake. By modelling these changes, the unintended consequences of public health policies can be predicted, such as how promoting plant-based diets may inadvertently decrease the intake of other beneficial food groups. A limitation of dynamic networks is the need for detailed longitudinal monitoring of diet over time; this often involves resource intensive data collection methods, such as repeated dietary recalls or food diaries over extended periods of time.

Hypergraphs extend traditional graph theory by allowing edges, known as hyperedges, to connect more than two nodes [40], making it possible to represent group-level interactions or clusters. Standard graph models only consider the pairwise interactions, such as edges between nutrients or foods, while hypergraphs can account for higher-order interactions. This ability to model interactions involving multiple nodes would be particularly useful in dietary pattern research as multiple nutrients often work together with shared function to influence health outcomes. For example, a hyperedge in a hypergraph could represent a meal containing protein, fats, and carbohydrates where the combined impact on health emerges from the interplay between these nutrients and cannot be explained by pairwise interactions alone. One limitation of hypergraphs is that their high computational demand can make them resource-intensive [41], especially for large datasets. Another limitation is that the complexity of hypergraphs often makes them hard to interpret [42], reducing their accessibility for researchers who are less familiar with advanced network methods.

Multilayered graphs represent systems with multiple interconnected layers, each capturing distinct but related interactions [43]. For example, one layer may represent nutrient interactions, another may represent food-level relationships, and another may represent food context. These graphs allow for a comprehensive exploration of both intra-layer and inter-layer connections [43]. Analysing cross-domain relationships is valuable in nutrition research as the impact of food context on nutrient intake can be examined. However, multilayered graphs are computationally demanding, especially for large datasets spanning multiple domains. They are also complex and can be challenging to interpret [43].

The choice of method depends on the research question, data type, and available computational resources. By leveraging these diverse tools, researchers can gain deeper insights into dietary patterns, overcoming some of the limitations of traditional dietary pattern analysis (Table 2).

In summary, it is possible that network approaches offer a more nuanced and comprehensive analysis of dietary patterns compared to traditional methods. Therefore, we aimed to (1) systematically map existing studies that have used network analysis in dietary research, and (2) establish guiding principles that can advance the methodological standards and promote more robust applications of these approaches in the field of nutrition research.

## 2. Methods

### 2.1. Study Design

This study was designed as a scoping review and was conducted in accordance with the PRISMA-ScR guidelines. The protocol was preregistered on Open Science Framework (https://doi.org/10.17605/OSF.IO/R5VE6).

### 2.2. Search Strategy and Selection Criteria

A comprehensive search was carried out using PubMed, Scopus, and PsycINFO to identify studies that applied network analysis to dietary pattern data in human populations. Searches covered the time period of inception to 7 March 2025. Studies were eligible if they (i) applied network models to dietary intake data, (ii) included human participants, and (iii) were written in the English language. Studies were excluded if network analysis was used for metabolomics or systems biology.

The following search terms were used for PubMed, Scopus, and PsycINFO: “network approach” or “network analysis” or “network method” or “graphical model” and “gaussian graphical model” or “GGM” or “mutual information network” or “mixed graphical model” and “dietary analysis” or “dietary data” or “nutrition analysis” or “food intake” or “diet” or “nutrition. Google Scholar was used to obtain additional articles identified by journal hand searching.

The core search terms (e.g., network analysis, graphical model) were chosen as high-level keywords to cast a wide net to capture any relevant study, even those using novel network algorithms. The few specific network models that were listed (GGM, MI, MGM) were included as they were already known to have been applied to dietary data, ensuring that the established methodologies within the field were not missed. To provide a thorough overview of the field and its potential, the introduction of this review discusses a wide range of network approaches, including those not yet applied to nutrition research (BNs, dynamic networks, hypergraphs, multilayered graphs).

### 2.3. Screening and Article Selection

**Figure 1:**
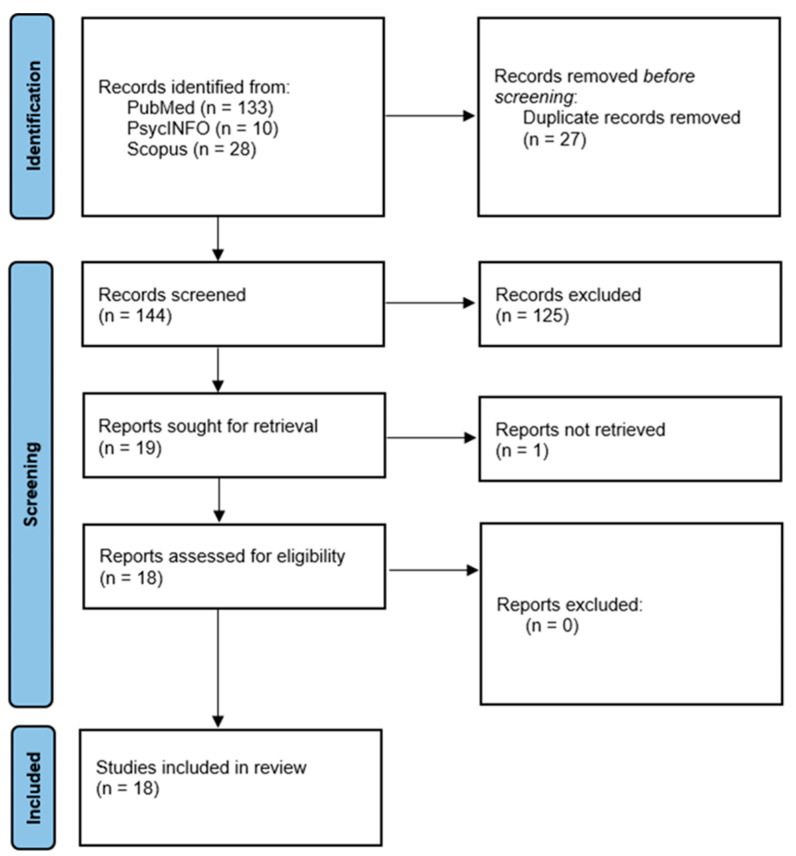
PRISMA flow diagram of literature screening and selection process.

All identified articles were imported into EndNote and duplicates were removed. Titles and abstracts were screened independently by two reviewers (R.M.J.T. and J.A.M.) against the predefined eligibility criteria. Potentially eligible full-text articles were then retrieved and assessed for inclusion. Full texts of the remaining articles were read to verify their suitability (R.M.J.T. and J.A.M.). Reference lists from the articles deemed suitable were checked for additional studies. Any discrepancies were resolved through discussion. The full screening and selection process is shown in Figure 1.

### 2.4. Data Extraction

Data were extracted into a standardised Excel spreadsheet by one reviewer (R.M.J.T.) and cross-checked by a second reviewer (J.A.M.) (available here https://osf.io/emd2q). Extracted information included author and year of publication, study aims, participant characteristics, dietary assessment methods, network model used, and appropriateness of the network model.

To evaluate the selected articles, we conducted a thematic analysis of methodological practices across the included studies, focusing on their alignment with best-practice recommendations for network analysis. Consistent with PRISMA-ScR and JBI guidance, we did not appraise risk of bias because our aim was to map methodological characteristics, not to evaluate intervention effects. Similarly, while general principles such as control of confounding, corrections for multiple comparisons, and outlier handling are critical for any dietary study, these data were not extracted as our focus was strictly on the methodological considerations specific to network analysis.

## 3. Results

### 3.1. Search and Selection of Network Studies

The search conducted in March 2025 identified 171 studies. After removal of duplicates, 144 unique articles remained to be screened. The screening of titles and abstracts against the predefined eligibility criteria (human dietary intake data, application of network analysis methods, English language) excluded 125 articles.

The remaining 19 articles were then assessed in detail using their full text. Of these, one was excluded as it did not have an English translation [44]. The remaining 18 articles were read, and all met the inclusion criteria leading to inclusion in the final review. The flow of studies from identification to final inclusion is represented in Figure 1.

### 3.2. Study Characteristics of Included Network Studies

The characteristics of the 18 studies included in this review are presented in Table 3. This table summarises each study’s population, dietary assessment method, network method, aims, and key findings. As detailed in the table, all studies were published between 2016 and 2024. Fourteen studies (78%) used food frequency questionnaires (FFQs), one used a flower-FFQ [45], two relied on 24 h recalls [46,47], and one used a Mediterranean diet adequacy questionnaire [48].

Nine studies analysed data from large, pre-existing cohorts: four from the Cancer Screening cohort in South Korea, three from the European Prospective Investigation into Cancer and Nutrition (EPIC) cohort, one from the Lifelines cohort, and one from the 3C study. The remaining nine studies used smaller, non-cohort-based samples. Sample sizes varied from 230 participants to 74,132 participants.

Regarding network approaches, eleven studies used GGMs, with one of these confirming their results with a semiparametric extension—semiparametric Gaussian copula graphical model (SGCGM). Two studies used only SGCGM. Three studies used MI matrices. Two studies used MGMs.

A wide range of health outcomes were analysed, highlighting the versatility of network approaches in dietary research. Cancer was the most frequently studied outcome (*n* = 5, including gastric and breast cancers). Other outcomes included incident prediabetes, metabolic syndrome, adiposity, obesity, non-alcoholic fatty liver disease, diet quality during pregnancy, anhedonia, dementia, and multiple sclerosis.

### 3.3. Objectives of Included Network Studies

The 18 included studies pursued a diverse range of objectives (Table 3), which can be grouped into two broad themes: descriptive mapping of dietary patterns and investigating associations with health outcomes.

Three studies were descriptive, aiming to characterise food co-consumption structures within populations. These populations varied, with one study looking at sex-specific dietary networks in German adults [49], one comparing meal-specific and habitual networks [46], and one mapping meal-level networks during pregnancy [47].

The remaining 15 studies investigated the associations between dietary networks and a variety of health outcomes. Cancer was the most frequently studied condition, with five investigations examining dietary networks in relation to gastric [50,51], breast [52], and overall cancer risk [53,54]. These included case–control studies identifying cancer-specific food consumption patterns [50,51,52].

A second cluster of studies centred on cardiometabolic health, where dietary networks were linked to measures such as general and abdominal adiposity [55], prediabetes [45], and metabolic syndrome [56,57]. In addition, several studies explored multimorbidity and broader chronic disease risk, applying network approaches to identifying the dietary structures associated with the development of multiple long-term conditions. For example, one study tested whether previously identified food-intake networks predicted major chronic diseases and their biomarkers [58], while another study used demographic and comorbidity data to reveal more complex interaction patterns [59].

Beyond these domains, network methods were extended to more specific outcomes. One study examined dietary networks in relation to non-alcoholic fatty liver disease [60], while the remaining three studies applied the approach in a neurological and mental health context. These included mapping diet patterns up to a decade before dementia onset [33], identifying food “hubs” in people with multiple sclerosis [48], and exploring the differences in networks between adults with and without anhedonia [61].

**Table 3:** Characteristics of the eligible studies.

Author (Year)	Population	Study Design	Dietary Assessment	Network Model	Aims	Findings
Slurink et al. (2023) [45]	74,132 participants, Lifelines cohort study	Prospective Cohort	Flower-FFQ	MGM	To investigate associations of total dairy and dairy types with incident prediabetes. To assess how dairy intake is linked with metabolic risk factors, lifestyle behaviours, and foods, as potential explanations for these associations.	Low fat milk intake associated with higher prediabetes risk. High-fat yogurt intake had nonsignificant inverse association with prediabetes risk. Associations may be confounded by behaviours relating to dairy intake.
Schwedhelm et al. (2021) [47]	365 women, 12 weeks gestation	Prospective Cohort	Three Automated Self-Administered 24 h dietary recalls	SGCGM	To investigate food networks across meals in pregnant women. To explore differences by overall diet quality classification.	Food combinations differed by meal and between dietary quality tertiles. **High diet quality group:** vegetables, whole-grain bread, cooked grains and nuts at breakfast. **Low diet quality group:** Sugar sweetened beverages, sandwiches, and fried potatoes at all main meals.
Felicetti et al. (2022) [48]	424 participants with MS, 165 healthy controls	Cross-Sectional	MeDi adequacy questionnaire	MI	To investigate food networks across meals in people with multiple sclerosis (PwMS) and healthy controls (HC). To explore differences by overall diet quality classification.	**PwMS Hubs:** Fruit, vegetables, cereal, and fish. **HC Hubs:** Meat and alcohol. PwMS showed overall healthier dietary pattern than HC.
Samieri et al. (2020) [33]	1522 participants (209 with dementia), 3C study	Nested Case–Control	FFQ	MI	To use network science to model complex diet relationships a decade before onset of dementia in a large French cohort.	Food networks substantially differed between cases and controls. **Cases:** Charcuterie was the main hub. **Controls:** Several disconnected subnetworks reflecting healthier choices.
Jayedi et al. (2021) [55]	850 participants	Cross-Sectional	FFQ	GGM	To describe dietary networks identified by GGM, representing patterns of dietary intake in a sample of Iranian adults. To investigate the potential associations of these dietary patterns with general and abdominal adiposity.	Identified 3 dietary networks: healthy, unhealthy, saturated fats. Saturated fats network was associated with a higher likelihood of central obesity. No association with general obesity.
Iqbal et al. (2016) [49]	27,120 participants, EPIC cohort	Cross-Sectional	FFQ	GGM (results confirmed through SGCGM)	To apply GGMs to derive sex-specific dietary intake networks representing consumption patterns in a German adult population.	**Men:** 1 major network including red meat, processed meat, and cooked vegetables. **Women:** similar network with addition of fried potatoes.
Schwedhelm et al. (2018) [46]	814 participants, EPIC cohort	Cross-Sectional	Three 24 h recalls	SGCGM	To estimate and describe meal and habitual dietary networks derived through SGCGMs. To compare relations found in meal networks to the ones present in the habitual network.	Meal-specific networks (breakfast, lunch, dinner) had distinct food communities. Meal-specific dietary network only partly reflected in habitual network.
Gunathilake et al. (2022) [54]	7477 participants (397 with cancer), Cancer Screening Cohort	Prospective Cohort	FFQ	GGM (also used PCA and RRR)	To investigate the association between dietary communities identified by a GGM and cancer risk.	A community composed of dairy products and bread was associated with a reduced cancer risk. In a matched population, poultry, seafood, bread, cakes and sweets, and meat byproducts showed significantly reduced risk of cancer.
Iqbal et al. (2019) [58]	22,245 participants, EPIC cohort	Prospective Cohort	FFQ	GGM (also used PCA)	To investigate the association between previously identified GGMs food intake networks and risk of major chronic diseases as well as intermediate biomarkers in the EPIC-Potsdam cohort.	A Western-type pattern was associated with increased risk of type 2 diabetes in women. A high-fat dairy pattern associated with lower risk of type 2 diabetes in both sexes.
Hoang et al. (2021a) [53]	10,777 participants (1049 with cancer), Cancer Screening Cohort	Cross-Sectional	FFQ	GGM	To identify major dietary patterns of Korean adults using a GGM. To examine the associations between dietary pattern (DP) scores and prevalence of self-reported cancer.	Identified 4 networks: principal, oil-sweet, meat, and fruit. Consumption of the oil-sweet pattern was lower in cancer patients, while meat and fruit pattern consumption was higher.
Jahanmiri et al. (2022) [56]	850 participants	Cross-Sectional	FFQ	GGM	To derive dietary networks and assess their association with metabolic syndrome.	Identified 3 networks: healthy, unhealthy and saturated fats. The saturated fats network was associated with high odds of metabolic syndrome.
Aguirre-Quezada and Aranda-Ramírez (2024) [57]	230 students	Cross-Sectional	FFQ	GGM	To apply GGMs to derived specific networks for groups of healthy and unhealthy obese individuals that represent the nutritional, psychological, and metabolic patterns in an Ecuadorian population.	Higher carbohydrate intake was associated with lower protein intake. For metabolically unhealthy obese individuals, intake of fibre, proteins, carbs, and fats was positively related to BMI.
Hoang et al. (2021b) [59]	7423 participants, Cancer Screening Cohort	Cross-Sectional	FFQ	MGM	To elucidate the complex interrelatedness among dietary intake, demographics, and risk of comorbidities.	Normal and heavy eating significantly associated with increased risks of elevated BP, hypertension, and mild kidney impairment.
Landaeta-Díaz et al. (2023) [61]	1242 participants	Cross-Sectional	FFQ	GGM	To explore food networks in the Chilean adult sample and in people with anhedonia symptoms.	Fruits, vegetables, and fast foods have central role in the main sample. In the anhedonia network, “pasta, rice and potatoes” and “bread” were more central.
Xia et al. (2020) [60]	2043 matched controls for 2043 newly diagnosed non-alcoholic fatty liver disease (NAFLD)	Case–Control	FFQ	MI	To construct dietary networks from network science. To explore the associations between complex dietary networks and NAFLD.	Dietary structures differed between groups. The case group had two major networks while the control group had one.
Fereidani et al. (2021) [52]	134 women with breast cancer, 266 hospital controls	Case–Control	FFQ	GGM	To compare food intake networks derived by GGMs for women with and without breast cancer to better understand how foods are consumed in relation to each other according to disease status.	Vegetables, fruits, sweets, and fried potatoes were central in both networks. The network of cases showed more conditional dependencies between foods than controls.
Gunathilake et al. (2020) [50]	415 gastric cancer cases, 830 controls, Cancer Screening Cohort	Case–Control	FFQ	GGM	To apply GGMs to identify dietary patterns. To investigate the associations between dietary patterns and gastric cancer risk in a Korean population.	Vegetable/seafood and fruit networks were associated with a decreased risk of GC. Highest tertile of vegetable/seafood score had a reduced risk of GC.
Gunathilake et al. (2021) [51]	268 patients with GC, 288 healthy controls	Case–Control	FFQ	GGM	To observe the combined effects of GGM-derived dietary patterns and the gastric microbiome on the risk of gastric cancer in a Korean population.	Vegetable/seafood pattern may interact with dysbiosis to attenuate the risk of GC in males. Dairy pattern may interact with dysbiosis to reduce GC risk in females.

### 3.4. Adherence to Methodological Best Practices

The remainder of this results section provides a thematic analysis of the included studies, evaluating their adherence to several key methodological best practices for network analysis (Table 4).

#### 3.4.1. Justifications for Using Network Models

The included studies justified their use of network analysis in several ways. The most common rationale was to counter the known limitations of traditional methods, a justification made by nine of the reviewed studies [46,47,52,53,55,56,57,59,60]. For instance, authors noted that network analysis was chosen because PCA can explain only a small proportion of the variability in food intake [47] and fails to demonstrate pairwise correlations between food groups [55].

Another common theme was the use of network analysis to complement existing research and gain additional insights into complex data [33,49,50]. One study framed its use as complementary without explicitly mentioning the limitations of traditional methods [48]. Two studies specifically focused on using network analysis to overcome the limitations of diet scores, allowing for an assessment of diet as a pattern rather than a sum of single food items [51,61].

Furthermore, three studies used network analysis directly alongside traditional dietary pattern analysis methods [45,54,58]. This was often performed to compare the dietary patterns identified by each approach, for example by using network analysis to derive dietary networks and then using PCA and reduced rank regression (RRR) for comparison [54], or by comparing new network-derived patterns to PCA patterns from the same cohort in an earlier study [58].

#### 3.4.2. Study Design and Causal Inference

Half of the reviewed studies utilised a cross-sectional design [46,48,49,53,55,56,57,59,61]. The nine remaining studies featured a mix of other observational designs – five were case-control [50,51,52,60], including one nested case-control study [33], and four were prospective cohort studies [45,47,54,58]. A crucial finding was that 17 out of 18 studies appropriately refrained from making strong causal claims from their observational data. Within the nine cross-sectional studies, authors were generally cautious; three studies explicitly stated that the design was the reason for this caution [48,56,59], and another acknowledged it as a limitation but did not specify why [55]. Only one study was identified as making causal inferences [54]. Although this study utilised a prospective cohort design, which is better suited for exploring temporal relationships than cross-sectional data, drawing causal conclusions from any observational design remains a significant challenge. This highlights the care that must be taken when interpreting associations, even from stronger study designs.

#### 3.4.3. Network Estimation and Regularisation

The reviewed studies employed several techniques to estimate their networks and control for spurious connections, with approaches varying by the chosen network model.

Among the studies using GGM-based models (GGMs, SGCGMs, and MGMs), LASSO regularisation was the most common approach, used in 14 of the 15 studies [45,46,47,49,50,51,52,53,54,55,56,57,58,59]. Only one GGM study did not employ a regularisation method [61]. However, the transparency in reporting the specific LASSO tuning parameter (λ) was inconsistent; just 8 of these 14 studies provided this detail [45,49,50,51,52,53,54,59], while one study explored network structures across a range of different tuning parameters (λ) [57].

For the three studies that used MI networks, one only applied thresholding to reduce network density [60], and one used only permutation testing to retain only statistically significant connections [48]. One study used both thresholding and permutation testing [33].

Regarding the stability and novelty of the findings, two studies, noted they were the first in their specific populations, and still made inferences from their findings [47,55].

#### 3.4.4. Use and Interpretation of Centrality Metrics

The application of centrality metrics to identify important foods or nutrients was inconsistent across the reviewed literature. Five of the eighteen studies avoided using centrality metrics in their analysis [47,48,49,52,60]. In contrast, the majority of studies (13 out of 18) did employ centrality metrics, and none of these studies acknowledged or discussed the potential limitations of this approach in the context of dietary network analysis [33,45,46,50,51,53,54,55,56,57,58,59,61]. For instance, one study examined node centrality using strength, betweenness, and closeness, ultimately opting to use the strength metric for its final analysis [45].

#### 3.4.5. Handling of Non-Normal Data

The studies employing GGMs used three distinct strategies to address the assumption of normally distributed data. The most robust approach was to use a nonparametric extension of the GGM; two studies used the SGCGM exclusively [46,47], and a third study used SGCGM to confirm the results of their primary GGM analysis [49].

The most common strategy was data transformation, with seven studies applying a log-transformation to their data to improve normality [49,50,51,52,53,54,58].

Finally, four of the GGM studies did not apply any correction for non-normal data. Of these, three acknowledged the issue as a limitation but did not address it [55,56,57], while one study did not acknowledge the limitation at all [61].

**Table 4:** Adherence of studies to the guiding principles of using network analysis for dietary pattern analysis.

Author (Year)	Justification for Using Network Models	Study Design and Causal Inference	Network Estimation and Regularisation	Use of Centrality Metrics	Handling of Non-Normal Data
Slurink et al. (2023) [45]	Used to aid interpretation of regression models. Holistic approach to aid traditional reductionist methods.	Did not attempt to make inferences about causality.	Used LASSO regularisation. λ value of 0.5 reported.	Uses centrality metrics. Did not discuss limitation.	N/A (used MGM).
Schwedhelm et al. (2021) [47]	Addressed limitations of PCA. Better alternative for revealing meal-specific food combinations.	Did not attempt to make inferences about causality.	Used LASSO regularisation. Did not report λ. Made inferences despite being the first study to test these associations.	Did not use centrality metrics.	Addressed via SGCGM. Excluded episodically consumed foods.
Felicetti et al. (2022) [48]	Used to see complex relations hidden in eating behaviour. Complementary to other research.	Acknowledged cross-sectional design prevents causal claims.	Used permutation testing.	Did not use centrality metrics.	N/A (used MI).
Samieri et al. (2020) [33]	Provided complementary information to other approaches. Gained additional insights into food-disease associations.	Did not attempt to make inferences about causality.	Used permutation testing. Used thresholding (edge weight >40).	Used centrality metrics. Did not discuss limitations.	N/A (used MI).
Jayedi et al. (2021) [55]	Addressed limitations of PCA.	Acknowledged cross-sectional design as a limitation but did not specify why.	Used LASSO regularisation. Did not report λ. Made inferences despite being the first study to test these associations.	Used centrality metrics. Did not discuss limitations.	Acknowledged the normality assumption. Did not apply any correction.
Iqbal et al. (2016) [49]	Limitations of existing methods of dietary pattern analysis warrant investigation of complementary approaches.	Did not attempt to make inferences about causality.	Used LASSO regularisation. λ value of 0.25 reported. Performed network stability analysis (bootstrapping).	Did not use centrality metrics.	Addressed via log-transformation. Confirmed GGM results with SGCGM.
Schwedhelm et al. (2018) [46]	Addressed limitations of traditional methods in understanding how patterns arise.	Did not attempt to make inferences about causality.	Used LASSO regularisation with cross-validation. Did not report tuning parameter (λ).	Used centrality metrics to assist interpretation. Did not discuss limitations.	Addressed by using SGCGM instead of GGM.
Gunathilake et al. (2022) [54]	Used GGM to derive dietary communities. Compared with PCA and RRR.	Used a prospective cohort design, made causal inferences.	Used LASSO regularisation. Optimal λ values reported, 0.32 and 0.34.	Used centrality metrics. Did not discuss limitations.	Addressed via log-transformation.
Iqbal et al. (2019) [58]	Used GGM to investigate diet-disease relationships. Reconstructed PCA patterns for comparison.	Did not attempt to make inferences about causality.	Used LASSO regularisation. Referred to previous publication for regularisation details.	Used centrality metrics. Did not discuss limitations.	Addressed via log-transformation.
Hoang et al. (2021a) [53]	Addressed limitations of PCA and RRR. Used GGM to resolve issues between methods.	Acknowledged cross-sectional design was not strong enough for causal claims.	Used LASSO regularisation. Optimal λ values reported (0.48, 0.52, 0.46).	Used centrality metrics. Did not discuss limitations.	Addressed via log-transformation.
Jahanmiri et al. (2022) [56]	Framed GGM as a “commanding method” compared to reductionist traditional techniques.	Acknowledged cross-sectional design prevents cause-and-effect conclusions.	Used LASSO regularisation. Did not report λ.	Used centrality metrics. Did not discuss limitations.	Acknowledged the normality assumption as a limitation. Did not apply any correction.
Aguirre-Quezada and Aranda-Ramírez (2024) [57]	Addressed limitations in previous studies analyses. Praised GGM for providing a comprehensive overview.	Did not attempt to make inferences about causality.	Used LASSO regularisation. Explored a range of λ rather than selecting one.	Used centrality metrics. Did not discuss limitations.	Acknowledged the normality assumption as a limitation. Did not apply any correction.
Hoang et al. (2021b) [59]	Addressed limitations of conventional approaches. Used network analysis to explore complex interactions.	Acknowledged cross-sectional design may not allow for a full investigation of causality.	Used LASSO regularisation. λ value of 0.5 reported. Assessed network accuracy via bootstrapping.	Used centrality metrics. Did not discuss limitations.	N/A (used MGM).
Landaeta-Díaz et al. (2023) [61]	Addressed limitations of diet scores. Used GGM to represent the underlying structure of food groups.	Did not attempt to make inferences about causality.	Did not use any regularisation techniques.	Used centrality metrics. Did not discuss limitations.	Did not acknowledge the normality assumption. Did not apply any correction.
Xia et al. (2020) [60]	Addressed limitations of traditional statistical methods. Used network methods to provide new insight.	Did not attempt to make inferences about causality.	Used thresholding (edge weight ≥ 0.30) for interpretability.	Did not use centrality metrics.	N/A (used MI).
Fereidani et al. (2021) [52]	Addressed limitations of existing methods. Used GGM to show how foods are consumed in different combinations.	Did not attempt to make inferences about causality.	Used LASSO regularisation. λ value of 0.3 reported.	Used a nonstandard definition of “central food groups”.	Addressed via log-transformation.
Gunathilake et al. (2020) [50]	Used as a complementary strategy for investigating diet-disease relationships.	Did not attempt to make inferences about causality.	Used LASSO regularisation. Optimum λ value reported (0.38).	Used centrality metrics. Did not discuss limitations.	Addressed via log-transformation.
Gunathilake et al. (2021) [51]	Assessed diet as a pattern rather than a sum of single food items.	Did not attempt to make inferences about causality.	Used LASSO regularisation. Optimum λ value reported (0.37).	Used centrality metrics. Did not discuss limitations.	Addressed via log-transformation.

### 3.5. Synthesis of Methodological Adherence

The methodological practices of the 18 included studies, detailed in Table 4, reveal a field with considerable promise but also significant inconsistencies. Most studies provided a clear rationale for employing network analysis, with the most common justification being to overcome the known limitations of traditional methods, such as PCA.

Despite strong justifications, several methodological challenges were prevalent across the literature. In terms of study design, half of the articles used cross-sectional data. While most of these studies appropriately refrained from making causal claims, the reliance on cross-sectional designs limits the field’s ability to move beyond identifying associations. Inconsistencies were also apparent in network estimation. Although LASSO regularisation was commonly used in the studies using GGMs to control for spurious connections, transparency in reporting was inadequate as 43% of these studies failed to provide specific details on the tuning parameter (λ) used, a practice that can limit reproducibility.

Furthermore, the application and interpretation of network metrics was an area of concern. Many of the reviewed studies (13 out of 18) employed centrality metrics to identify key dietary components, yet none acknowledged or discussed the potential limitations of this approach in the context of unbounded dietary networks. Finally, the handling of non-normal data varied considerably among studies using GGMs. While a few studies used robust nonparametric extensions such as the SGCGM, the most common strategy was data transformation, and three studies did not apply any correction for normality.

Collectively, these findings highlight the need for greater methodological standardisation, which the guiding principles proposed in this review aim to address.

## 4. Discussion

This scoping review provides the first comprehensive map of the emerging field of dietary network analysis, revealing its rapid growth and versatile application across a range of health outcomes. However, our thematic analysis of the 18 included studies also identified a consistent pattern of methodological issues, most notably a reliance on cross-sectional data, inconsistent reporting of estimation parameters, and the uncritical use of centrality metrics. The prevalence of these challenges suggests a critical need for methodological standardisation.

### 4.1. Guiding Principles for Future Research

**Figure 2:**
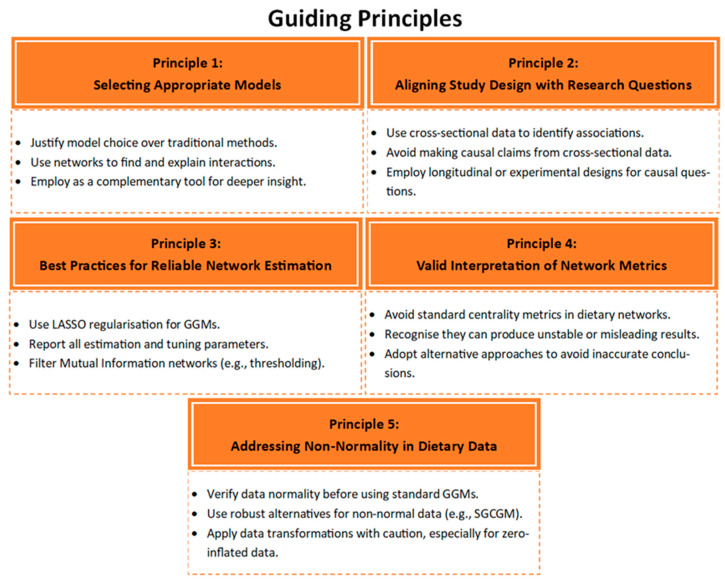
Five guiding principles for research using network approaches to dietary pattern analysis.

To address these limitations, we developed five guiding principles by synthesising the methodological gaps identified in our review with established best-practice recommendations from the wider network science literature. These principles, summarised in Figure 2, have been distilled into a new CONSORT-style reporting standard—the Minimal Reporting Standard for Dietary Networks (MRS-DN)—provided in the Supplementary Materials. The remainder of this discussion will detail each of these principles, which are designed to enhance the rigour and reliability of future research in this field.

#### 4.1.1. Principle 1: Selecting Appropriate Models

Researchers should only use network analysis when it addresses specific limitations of traditional multivariate methods or provides complementary insights that align with the research question [62]. Using network analysis to counter the limitations of widely used dietary pattern analysis methods and to complement the data obtained by these methods is effective in building on the existing knowledge to uncover interactions that have previously been concealed. It is critical, however, to ensure that network analysis is the most appropriate method for the research question at hand.

#### 4.1.2. Principle 2: Aligning Study Designs with Research Questions

Aligning the study design with the research question is a foundational principle of epidemiological research. Accordingly, studies using cross-sectional data are appropriate for identifying associations but should not be used to infer causality, as this can lead to misleading conclusions about the relationship between diet and health. This distinction is critical, as it is a basic tenet that cross-sectional data can only establish correlation, not causation [62]. These inappropriate claims risk misinforming the scientific community and may potentially lead to flawed public health recommendations based on evidence which can only support an association. For instance, if a cross-sectional study finds that individuals who consume more olive oil have a lower prevalence of heart disease, it is a methodological error to conclude that olive oil causes a reduction in heart disease. The observation could be confounded by other factors (e.g., individuals using olive oil may be more likely to consume more vegetables, exercise regularly, or have high socioeconomic status). A flawed causal claim could lead to public health advice that overemphasises a single ingredient, when the real benefit lies in a broader, healthier lifestyle pattern that remains unobserved.

For causality-focused research questions, longitudinal data may be better suited. Where longitudinal dietary data are available, time-varying network models—such as dynamic or multi-level vector autoregression (mlVAR) frameworks—can reveal how food-co-consumption patterns evolve and exert lagged effects on health, providing richer causal insight; however, they should be applied only when there are enough repeated recalls or diary days to capture true day-to-day variation, and with careful handling of compositional constraints, seasonality, and measurement error to avoid artefactual dynamics.

#### 4.1.3. Principle 3: Best Practices for Reliable Network Estimation

The robustness of estimated networks can be questionable, as networks may appear similar in their global characteristics while their detailed characteristics vary substantially [62,63]. Therefore, inferences should not be drawn before results have been rigorously replicated [62]. To create more reliable and interpretable networks, specific best practices should be followed.

When using GGM-based methods, robust methods such as LASSO regularisation should be used to minimise spurious connections, as this technique limits the number of spurious edges to obtain more interpretable networks [64]. Instead of just noting that regularisation was used, it is helpful to specify (1) the exact method (e.g., graphical LASSO), (2) the tuning parameter chosen (e.g., λ = 5), and (3) the criterion for selecting that parameter (e.g., extended Bayesian information criterion, with the hyper-parameter γ). This extra detail makes the analysis easier for others to interpret and replicate [64]. Similarly, when using MI networks, techniques like thresholding (retaining edges above a certain value) and permutation testing (recalculating mutual information on randomised data to keep only significant connections) should be used to create a more robust network with fewer spurious edges [65,66].

Before interpretation, the stability of the final network structure should be rigorously assessed. A common and effective method is bootstrapping, where the network estimation procedure is repeated on numerous subsamples of the data to see if the structure remains consistent [67]. Interpreting a network without first confirming its stability can lead to conclusions based on sample-specific noise rather than a robust underlying pattern.

Overall, the minimum that studies should be reporting is (1) the software and packages used for the analysis, (2) the exact regularisation method, (3) the criterion for parameter selection, (4) the final value(s) of the parameters (e.g., λ, γ), and (5) the results of a stability analysis for the networks edges.

#### 4.1.4. Principle 4: Valid Interpretation of Network Metrics

Researchers should carefully interpret centrality metrics (e.g., degree, closeness, betweenness) for dietary networks. Most network centrality metrics were developed for bounded networks, where the nodes are clearly defined and fixed [62]. Dietary pattern analysis, however, involves what are known as unbounded networks: dynamic systems that, unlike bounded networks, do not have a fixed number of nodes or connections. When centrality metrics are applied to unbounded networks, their interpretability is compromised, making them unstable and unreliable [62]. This can lead to inaccurate conclusions. For example, one study concluded that “a low centrality predictability of low-fat milk intake in the networks may indicate that significant associations in the regression analysis could be due to intake levels coinciding with influential risk factors” [45]. However, given the limitations of centrality metrics in unbounded networks, this conclusion may not be valid. While the choice of the “strength” metric in that study was based on its relative stability, this does not negate the broader issue that these metrics can produce misleading information [68]. For example, if “white rice” appears as the most central food in a dietary network from an Asian population, it might be wrongly interpreted as the single most important driver of the overall dietary pattern. However, its high centrality may simply be an artifact of it being a staple food consumed frequently with many different, otherwise unrelated, food groups (e.g., fish, vegetables, meat). A more robust analysis of the network’s community may reveal distinct “fish and rice” and “vegetable and rice” patterns, providing a more nuanced and accurate picture of the diet that is not apparent from the simple node ranking. Therefore, researchers might consider either avoiding centrality metrics entirely or adopting alternative approaches that account for the unbounded nature of dietary networks.

If centrality metrics are calculated, then their stability must be assessed as a minimum requirement (e.g., using a case-dropping bootstrap procedure). However, even stable centrality metrics should be interpreted with extreme caution.

A more robust alternative to single-node centrality is to identify and analyse the network’s community structure [69]. This approach focuses on broader co-consumption patterns, which are often more stable and interpretable than the importance of a single food item.

Furthermore, in dietary graphs that evolve over time, centrality can reflect exposure and catalogue growth rather than true structural influence [62]. When centrality is reported, treat it as descriptive, pair it with stability checks (e.g., case-dropping bootstrap), and interpret cautiously [68]. Alternatives include growth-robust diagnostics: (i) community/module stability across time-slices (e.g., adjusted Rand/NMI), (ii) permutation-based information flow that compares simulated reach to degree/strength/time-matched nulls, (iii) bipartite or hypergraph formulations for many-to-many relations (product–ingredient, product–retailer), and (iv) motif persistence that tracks recurring higher-order patterns [69]. This avoids over-interpreting single-node centrality claims such as the “low centrality predictability” example in prior work [45]. For changing product lists, analyse in fixed windows, report null-relative effect sizes, and summarise each node by a compact tuple (excess flow, module persistence, motif recurrence) with uncertainty.

#### 4.1.5. Principle 5: Addressing Non-Normality in Dietary Data

Dietary data is rarely normally distributed, often being skewed and zero-inflated (i.e., containing many “no consumption” reports), violating a core assumption of standard GGMs [70]. When preprocessing the data, the distribution of the dietary data should be examined and reported. Simple log-transformations are often insufficient for zero-inflated data [71], and adding a small constant to avoid errors with zero values can distort the data, leading to spurious connections and complicating the interpretation of the results [72]. To illustrate, consider two healthy but infrequently consumed foods, such as “sardines” and “flaxseeds”, that are rarely eaten together. In a dataset with many zero values for these items, the common practice of adding a small constant before applying a log-transformation can artificially shrink the data’s variance and create a spurious positive correlation between them. A subsequent network analysis might then incorrectly identify a “health-conscious” dietary pattern of “sardines and flaxseeds” when no such co-consumption pattern exists in the population. Using a model appropriate for non-normal data like an SGCGM would correctly reveal the absence of a direct connection. The method for handling zero values must be explicitly described and justified.

When data is not normally distributed, standard GGMs should be avoided. Researchers should instead opt for a more robust alternative. The SGCGM is a nonparametric extension of the GGM which is designed for skewed data, making it a superior choice in such cases. Alternatively, researchers could use methods which do not assume normality, such as an MI network or an MGM.

The minimum that studies should be reporting is (1) the distributional properties of their dietary data, (2) the specific method used to handle zero values, and (3) a clear justification for the chosen network model considering the data’s distribution (e.g., why a standard GGM was used for non-normal data).

When deciding between SGCGM and MI/mixed-model residual networks, authors should consider the following. (1) Zero inflation: If zeros are mainly *structural* (true absence) and exceed roughly 40%, treat the data in two parts: model presence/absence first (logistic or hurdle/ZINB) and build a residual network on the positive counts; this favours an MI/mixed-model route because the zero process is explicitly modelled. When zeros are mostly *sampling artefacts* and the prevalence of each node exceeds about 5%, apply a small, prevalence-aware additive smoothing (ε) and run a brief ε-sensitivity check; under that scenario a rank-based SGCGM is suitable because it handles the remaining non-normality without further transformation. (2) Interpreting edge weights after transformation: With an SGCGM, the partial edges refer to *conditional rank associations* on a latent Gaussian scale—direction and strength show whether two foods tend to rise or fall together once all others are held constant, independent of original units. In an MI/mixed-model residual network the edges capture conditional associations in the portion of each variable that the mixed model has *not* explained; their meaning therefore sits on whatever scale the outcome was modelled (e.g., log counts, centred proportions). State this scale explicitly and, where helpful, translate one edge back to the original units in a worked example so readers see how the transformation affects interpretation.

### 4.2. Future Directions

Future dietary network research should engage more deeply with the temporal dynamics inherent to nutritional epidemiology. The current literature is dominated by cross-sectional data, which only provides a static snapshot of diet. However, dietary patterns are not static; they evolve over an individual’s lifespan due to a variety of factors, including population ageing, changes in health status, or shifts in socioeconomic status [25].

To capture these crucial long-term dynamics, future studies can use advanced network models which are well-suited for longitudinal data. Dynamic networks can explicitly model how food co-consumption patterns change over time, allowing researchers to track dietary shifts within populations in response to interventions or health changes [28,38]. Similarly, multilayered graphs offer a powerful way to integrate dietary data with other evolving domains, such as health biomarkers or physical activity, providing a more holistic view of the diet-health relationship across the lifespan [43].

A significant opportunity for future research lies in applying BNs to dietary data as this has not yet been performed. These networks are able to represent relationships through directed graphs, allowing researchers to explore potential causal pathways [34], a major step forward for a field dominated by cross-sectional data. As previously mentioned, BNs could be used to model directional phenomena, such as the fat-sugar seesaw, where a reduction in fat may lead to a subsequent increase in sugar intake [35].

Ultimately, if dietary network research is to inform robust public health recommendations and policy, future studies must move beyond cross-sectional snapshots and invest in longitudinal and interventional designs that can truly unravel causal relationships within evolving dietary patterns.

Another promising direction is the use of hypergraphs to move beyond the simple pairwise connections between foods, which also has not yet been performed. Standard network graphs can only represent an edge between two nodes at a time, while hypergraphs can use hyperedges to connect a group of three or more nodes simultaneously to represent a meal or synergistic combination of nutrients [40]. This allows for the modelling of higher-order interactions, where the health impact of a combination of foods cannot be explained by its individual components alone, potentially providing a much deeper understanding of the complex food synergies crucial for health.

### 4.3. Strengths and Limitations of the Current Work

A key strength of this review is its systematic and rigorous methodology. We conducted a comprehensive search across multiple relevant databases and followed the PRISMA-ScR guidelines for scoping reviews. The inclusion of two independent reviewers for the study selection process minimises the risk of bias and enhances the reliability of our findings. Furthermore, this review moves beyond a simple summary of the literature. By critically evaluating the included studies against an established framework of best practices and generating a novel set of guiding principles, this work provides a tangible roadmap for future researchers in this emerging field.

Despite these strengths, several limitations should be acknowledged. First, as a scoping review, our aim was to map the field rather than conduct a formal quality assessment or risk-of-bias analysis for each individual study; therefore, the quality of the primary studies was not formally appraised. Second, our review may be subject to publication bias, as studies with null or nonsignificant findings may be underrepresented in the published literature. Additionally, our search was limited to English-language publications; however, only one relevant study was excluded because no English translation was available. Finally, dietary network analysis is a rapidly evolving field. While our search was comprehensive up to its cut-off date, new studies will have been published in the interim. A further limitation, inherent to the primary studies included in this review, is the potential for measurement error from dietary assessment methods. The analysis revealed a heavy reliance on FFQs, which were used in 78% of the reviewed studies. While FFQs are effective for capturing long-term dietary patterns, they are susceptible to systematic errors, including recall bias [11] and social desirability bias [73]. The few studies that used 24 h recalls may have more accurate data for a given day but might not capture a participant’s habitual diet [74]. The choice of assessment tool inevitably influences the data structure, and these potential sources of measurement error should be considered when interpreting the resulting dietary networks.

One strength of the proposed guiding principles is that they provide an accessible framework for a field that is currently marked by significant methodological heterogeneity. By translating complex analytical issues into an actionable reporting standard—the Minimal Reporting Standard for Dietary Networks (MRS-DN)—this work can help to standardise practices and improve the quality of future research. However, this proposal has limitations. Dietary network analysis is a rapidly evolving field, and the MRS-DN is intended as a living document that will be updated iteratively as new computational methods and statistical techniques emerge. Furthermore, these principles are intended as focused guidance for network analysis and should be adapted to the unique characteristics of each dataset and used alongside broader reporting guidelines as appropriate.

### 4.4. Conclusions

Given that current approaches to dietary pattern analysis fail to adequately capture nutritional complexity, the objective of this scoping review was to systematically map and synthesise studies applying network analysis to dietary data and to establish guiding principles that advance methodological rigour in future research.

The primary contribution of this scoping review is the establishment of the evidence-based, methodological framework for the use of network analysis in dietary research. While previous studies have applied network models to nutritional data, this review is the first to systematically map the landscape of this emerging field, critically synthesise its recurrent methodological challenges, and translate these findings into five guiding principles, presented as a CONSORT-style checklist—the Minimal Reporting Standard for Dietary Networks (MRS-DN) (Supplementary Materials). By moving beyond a simple summary of the literature to provide a tangible roadmap for future researchers, this work addresses a critical gap and provides the foundational guidance needed to improve the rigour, reproducibility, and reliability of studies in this promising area. Ultimately, the adoption of these principles will help researchers to harness the full potential of network analysis to advance understanding of the complex relationship between diet and health.

## Figures and Tables

**Table 1:** Traditional methods for dietary analysis.

Method	Algorithm	Linear/Nonlinear	Assumptions	Strengths/Limitations
Principal Component Analysis (PCA)	Eigenvalue decomposition	Linear	Assumes normally distributed data, linear relationships between variables, uncorrelated components.	Identifies what dietary patterns exist in a population. Can determine which foods are consumed together in a diet but does not reveal interactions between those foods.
Factor Analysis	Factor extraction	Linear	Assumes normally distributed data, linear relationships, data can be grouped into latent factors.	Can identify the underlying dietary factors that explain variations in food intake. However, does not provide information about how particular foods interact.
Cluster Analysis	k-means, hierarchical clustering	Nonlinear	Assumes defined clusters with similar characteristics and independent observations.	Groups individuals based on their dietary patterns. Useful for segmenting consumers based on dietary patterns. Can handle nonlinear associations between variables. Assumes pairwise similarity or proximity but does not explicitly capture direct or indirect interdependencies among multiple variables.
Dietary Index/Scores	Predefined scoring	Linear	Assumes each score represents healthfulness, often based on a reference diet. Each component is typically weighted (sometimes equally), ignoring potential interactions between components. Requires prior knowledge.	Can identify how closely an individual’s diet aligns with a healthy/reference dietary pattern.

**Table 2:** Network methods for dietary analysis.

Method	Algorithm	Linear/Nonlinear	Assumptions	Strengths/Limitations
Gaussian Graphical Models (GGMs)	Inverse covariance matrix estimation	Linear	Assumes normally distributed data, linear relationships, requires sparsity.	Measures the conditional dependencies between different foods. Reveals how certain foods are commonly consumed together, or how foods may displace each other in the diet. Can increase understanding how variables (e.g., foods, nutrients) directly interact, independent of others in the context of the whole diet. Relies on partial correlation matrix and is sensitive to non-normally distributed data.
Mixed Graphical Models (MGMs)	Combination of GGM and discrete modelling techniques	Both	Assumes mixed data types can be represented in a joint network, requires sparsity.	Can identify direct relationships while accommodating diverse variable types. Standard MGMs assume linear relationships but with extensions such as kernel methods nonlinear models can be developed.
Mutual Information Network	Information-theoretic methods	Nonlinear	No strict distributional assumptions, assumes mutual information represents dependence.	Uses entropy-based measures to quantify shared information. Reveals how certain foods are commonly consumed together, even in nonlinear relationships (e.g., nutrient thresholds or diminishing returns). Similar to GGM but without normality assumption. Does not differentiate direct and indirect associations.
Bayesian Networks (BNs)	Directed acyclic graphs	Both	Assumes probabilistic relationships between variables.	Provides insights into causality and allows the exploration of causal pathways. Can incorporate prior knowledge for enhanced interpretability. Computationally intensive when discovering unknown networks.
Dynamic Networks	Time-varying graph algorithms	Both	Requires longitudinal data with high temporal resolution.	Models time-varying dietary patterns and tracks changes in diet over time. Useful for predicting unintended consequences of interventions. Requires resource-intensive longitudinal data collection for accurate analysis.
Hypergraphs	Hyperedge-based graph algorithm	Both	Assumes interactions can involve more than two nodes.	Captures higher-order interactions. Useful for modelling the combined health impact of foods/nutrients which are unable to be explained by pairwise interactions. Computationally demanding and resource intensive. Complexity may affect interpretability.
Multilayered Graphs	Layered network construction	Both	Assume information is shared between all layers.	Enables analysis of intra- and inter-layer connections. Valuable for cross-domain analysis. Computationally demanding and complex. Challenging to interpret for large datasets.

## Data Availability

Preregistration and data extraction are available at: https://osf.io/emd2q.

## Supplementary Materials

The following supporting information can be downloaded at: https://www.mdpi.com/article/10.3390/nu17203261/s1, File S1: The Minimal Reporting Standard for Dietary Networks (MRS-DN).

## Abbreviations

The following abbreviations are used in this manuscript:

DASH	Dietary approach to stop hypertension
PCA	Principal component analysis
GGM	Gaussian graphical model
MGM	Mixed graphical model
MI	Mutual information
BN	Bayesian networks
EPIC	European Prospective Investigation into Cancer and Nutrition
SGCGM	Semiparametric Gaussian copula graphical model
RRR	Reduced rank regression
MeDi	Mediterranean diet
PwMS	People with multiple sclerosis
HC	Healthy controls
