# Supplementary material for: Unveiling Dietary Complexity: A Scoping Review and Reporting Guidance for Network Analysis in Dietary Pattern Research
Source: Nutrients. 2025 Oct 17;17(20):3261. doi: 10.3390/nu17203261 (PMC12567371; doi:10.3390/nu17203261)
Supplement: Supplementary file 1 [file nutrients-17-03261-s001.zip › nutrients-3825379-supplementary.pdf]

Minimal Reporting Standard for Dietary Networks (MRS-DN).

Purpose. This checklist outlines the Minimal Reporting Standard for Dietary Networks (MRS-DN). It is intended for authors, reviewers, and editors to ensure that studies are reported with sufficient clarity and transparency to allow for critical assessment and replication. Adherence to these standards aims to improve the methodological rigour and reliability of future research in this field. As this is a rapidly evolving area, this checklist will be updated iteratively as new methodological and statistical approaches develop. The MRS-DN is designed to complement, not replace, other broader reporting guidelines (e.g., STROBE, CONSORT-NUT), and should be used alongside other methodological best practices as appropriate.

1) Data & Study Design

- **Dietary Instrument:** Report the full name of the dietary assessment tool (e.g. FFQ, 24HR) and provide a citation for its validation in a relevant population.
- **Assessment Period:** State the time window and recall period of the instrument (e.g. 7-day FFQ, 2x24HR).
- **Study Design:** Specify study design (e.g. cross-sectional, prospective cohort, intervention). Avoid making causal claims unless the design explicitly supports them.
- **Population:** Describe the participant sample. Include number, age, sex, country, cohort.

2) Model Choice

- **Justification:** Justify choice of network class (GGM/SGCGM/MGM/MI/BN/other) based on the data properties (continuous/categorical/mixed), distribution, and research questions.
- **Advanced Representations:** If using bipartite or hypergraphs, state the construction method (e.g. meal x food to food x food projection) and discuss any caveats.

3) Estimation & Selection Details

- **Software:** Report the software and specific packages (including version numbers) used for the analysis.
- **Estimation Method:** State exact regularisation and estimation method used (e.g. EBICglasso, permutation MI)
- **Parameter Selection:** Describe the selection criterion (e.g. EBIC) and report all relevant settings (e.g. EBIC-γ)
- **Tuning Values:** Report final tuning parameter values (e.g. λ, γ) or equivalent thresholds. If a grid search was used, report the range of values searched.

4) Stability & Robustness

- **Edge Stability:** Report the results of a bootstrap or sampling analysis to demonstrate the stability of the network's edges (e.g. provide edge selection frequencies/confidence intervals).
- **Community Stability:** If communities are a key part of the interpretation, report an analysis of their stability across resamples (e.g. variation-of-information, adjusted Rand index).
- **Alternative Checks:** Describe any other robustness checks performed (e.g. permutation influence analyses, replicate models in SGCGM/MI)

5) Data Distribution & Zero Handling

- **Data Properties:** Report descriptive statistics for each variable, including measures of distribution (e.g. skew, kurtosis) and the percentage of zero values.

- **Strategy for Zeros:** State the explicit strategy used to handle zero-inflated data (e.g. using preprocessing steps, using model which handles zeros naturally like SGCGM/MGM/MI). Avoid adding a small constant to enable log-transformation; if this method is used, it must be robustly justified, and its impact on interpretation must be discussed.
- **Transformations:** Describe any data transformations applied and discuss how they affect the scale and interpretation of the final edge weights.

6) Interpretation & Reporting

- **Interpretation Focus:** Primary interpretation should focus on robust, meso-scale structures like communities, modules, or motifs rather than single-node rankings. If centrality metrics are reported, their stability must be assessed, and clear caveats regarding their use in unbounded networks must be included.
- **Contextualisation:** Link the identified network patterns to meaningful dietary constructs (e.g. meals, cuisines, established food groups) rather than isolated nodes.

7) Transparency & Reproducibility

- **Open Materials:** Provide a link to all materials necessary to reproduce the analysis, including analysis code and summary data, in a public repository (e.g. OSF, Zenodo).
- **Preregistration:** State whether the study was preregistered and provide a link. Report and justify any deviations from the preregistered plan.

The Minimal Reporting Standard for Dietary Networks (MRS-DN)

Item #	Recommendation	Reported on Page #
**Data & Study Design**		
**1**	Dietary instrument named and validation cited; assessment period stated.	
**2**	Study design stated.	
**3**	Participant population described.	
**Model Choice**		
**4**	Network class justified against data properties.	
**Estimation & Selection Details**		
**5**	Software, packages, and version numbers reported.	
**6**	Estimation method, parameter selection criteria, and final tuning parameters reported.	
**Stability & Robustness**		
**7**	Stability of network edges and/or community structures assessed and reported.	
**Data Distribution & Zero Handling**		
**8**	Descriptive statistics for each variable reported.	
**9**	Explicit strategy for handling zero values reported and justified.	
**10**	Impact of data transformations on edge interpretation discussed.	
**Interpretation & Reporting**		
**11**	Interpretation focused on robust structures; if using centrality metrics, caveats and stability reported.	
**Transparency & Reproducibility**		
**12**	Public link to analysis code and/or data is provided.	
**13**	Preregistration is stated, and a link is provided.	
